# Advancing Global Health Security through Rapid Operational Research on Diagnostics during Outbreaks

**DOI:** 10.4269/ajtmh.24-0213

**Published:** 2025-04-01

**Authors:** Ezekiel Boro, Anne Hoppe, Daniel G. Bausch

**Affiliations:** ^1^ FIND, Geneva, Switzerland;; ^2^ Liverpool School of Tropical Medicine, Liverpool, United Kingdom;; ^3^ Elizabeth Glaser Pediatric AIDS Foundation, Geneva, Switzerland

Global health security (GHS) relies on accurate and timely diagnosis of pathogens with epidemic and pandemic potential, as well as effective and timely interventions.^1^ Operational research (OR), defined as “research into strategies, interventions, and tools or knowledge that can enhance the quality, coverage, and effectiveness or performance of the health system or program in which the research is being conducted,” plays a crucial role in advancing GHS worldwide.^2^ Operational research conducted in low-and middle-income countries (LMICs) helps bolster GHS by ensuring that global health policy and programmatic recommendations, strategies, and interventions, which are often developed based on insights and findings from high-income countries, are appropriately adapted and relevant to LMICs.^3^

At the height of the COVID-19 pandemic, many LMICs had very poor COVID-19 testing rates despite the early availability of quality-assured laboratory-based and point-of-care diagnostic tests. For instance, as of June 2022, almost two and a half years after the World Health Organization declared COVID-19 a public health emergency of international concern, and almost two years after they first granted emergency use listing to a SARS-CoV-2 rapid lateral-flow test, only 0.4% of the 3 billion COVID-19 tests reported globally were performed in low-income countries.^4^ Testing rates in LMICs were grossly inadequate for effective COVID-19 surveillance on virtually any level, national or local.

As commercial COVID-19 tests began to roll out, first in high-income countries and then later in LMICs, it became clear that one particular diagnostic platform, antigen rapid diagnostic tests (Ag-RDTs), held the potential to revolutionize disease surveillance during outbreaks, allowing broad access to diagnostics on both individual, and population levels never seen before. Furthermore, COVID-19 surveillance using Ag-RDTs was potentially “customizable,” and adaptable to diverse social, cultural, and economic settings around the world, including on a local community level, such as in schools, workplaces, and places of worship, and at mass gatherings. However, while this new tool provided enormous potential, it also posed a new challenge—determining the practical, implementable, and effective use case for Ag-RDTs in each specific setting. A clear and critical need emerged for rapid OR to provide local, regional, and global health authorities and policymakers the evidence base and practical knowledge to inform guidelines for the use of Ag-RDTs to prevent SARS-CoV-2 transmission.

To this effect, in 2022 the international organization FIND (Geneva, Switzerland) under the auspices of the Access to COVID-19 Tools Accelerator (ACT-Accelerator) commissioned 17 OR studies in 13 countries across Africa, the Caribbean, and Asia to explore community-based applications of Ag-RDTs for SARS-CoV-2.^5^ Resources were allocated via a competitive peer-reviewed process, calling for and prioritizing rapid evidence gathering for actionable steps for outbreak control, with study implementation limited to six months and funds available for less than 12 months.^6^ In this supplement of the *American Journal of Tropical Medicine and Hygiene*, we share actionable findings and insights from these studies, as well as a final piece summarizing key success factors, challenges, and recommendations resulting from the implementation of this global OR collaborative on diagnostics.

These locally driven studies supported the development of effective Ag-RDT testing strategies aimed at early detection and prevention of virus transmission within schools and universities, workplaces, border communities, markets, mobile populations, and marginalized communities. They provided insights about effectiveness, feasibility, and acceptability of different community-based testing strategies, often in conjunction with other control measures, such as awareness and advocacy campaigns to dispel myths about COVID-19 and SARS-CoV-2 testing, nonpharmaceutical interventions such as physical distancing, handwashing, provision of alcohol-based hand gels and face masks, and control with self-isolation and quarantine. Some assessed the financial cost drivers of community-based testing with Ag-RDTs. Where appropriate, testing was linked to COVID-19 vaccination campaigns.

The experiences and contributions of the many FIND/ACT-Accelerator’s rapid diagnostics OR initiative study partners during the COVID-19 pandemic highlight the important role of OR in advancing GHS. This collaboration rose to tackle some of the diagnostic testing challenges posed by the pandemic by working with partners in LMICs to swiftly identify and address pertinent evidence gaps, and to provide research-driven responses. By deploying resources to investigate use of rapid testing strategies and technologies, the initiative showcases the agility and adaptability required in crisis situations.

The studies also demonstrate some valuable practical lessons regarding implementation of OR during epidemics; for many studies and regions, the rapid evolution of the COVID-19 pandemic, often with waxing and waning transmission, constituted a moving target, posing considerable practical impediments to study implementation. Many studies had the “bad luck” of low SARS-CoV-2 transmission rates during their implementation, with insufficient cases to reach the necessary statistical endpoints to assess effectiveness of the studied intervention. They nevertheless provide valuable information with regard to study design, feasibility, and acceptability. These and other valuable lessons from this global OR collaborative on diagnostics, including key success factors, challenges, and recommendations to optimize OR during future outbreaks, are discussed in detail in the supplement’s concluding manuscript.

Operational research is foundational to advancing GHS, particularly in the context of diagnostic interventions during pandemics. FIND/ACT-Accelerator’s rapid OR diagnostics initiative exemplifies the critical role of OR in responding promptly to emerging health threats. However, challenges persist in conducting rigorous research amid rapidly evolving situations in low-resource settings, with enormous GHS gaps and competing priorities, leading to difficulties in establishing diagnostic effectiveness conclusively. An OR framework should be integrated into GHS initiatives, such as the 100 Days Mission, to ensure that the resulting diagnostic, therapeutic, and vaccine products are both effective and suitable for and acceptable to populations in LMICs.^7^^,^^8^ As the world continues to grapple with health crises, refining OR methodologies and emphasizing adaptive approaches will be essential to fortify our defenses against future threats.
